# Co-stimulation Therapy in Rheumatoid Arthritis: Today and Tomorrow

**DOI:** 10.1007/s40674-015-0029-0

**Published:** 2015-09-29

**Authors:** Michael Schiff

**Affiliations:** Department of Rheumatology, University of Colorado, 5400 South Monaco Street, Greenwood Village, Denver, CO 80111 USA

**Keywords:** Rheumatoid arthritis, Abatacept, Co-stimulation, T cell therapy, CTLA-4-Ig

## Abstract

Abatacept is the only T cell co-stimulation modulator approved thus far for the treatment of moderate-to-severe rheumatoid arthritis (RA) and is licensed for use in patients with an inadequate response to methotrexate (MTX) and/or anti-tumor necrosis factor (anti-TNF) therapy. The upstream mechanism of action of abatacept leads to downstream effects in a variety of cell types associated with the production of autoantibodies and pro-inflammatory cytokines implicated in RA. Accumulating data also suggest effects on other cells involved in the pathogenesis of RA, including regulatory T cells and osteoclasts. Clinical trials have demonstrated that abatacept is an effective and well-tolerated treatment in RA. More recently, evidence from the *A*ssessing *V*ery *E*arly *R*heumatoid arthritis *T*reatment (AVERT) trial showed that complete drug-free remission following treatment with abatacept may be a possibility in some patients with early RA, indicating that the disease course could be altered by early intervention. Equivalent efficacy and onset of action of abatacept and anti-TNF therapy have also been demonstrated in patients with an inadequate response to MTX in the *A*batacept versus adali*M*umab com*P*arison in bio*L*ogic-naïv*E* rheumatoid arthritis subjects with background methotrexate (AMPLE) trial. Together, these findings support the use of abatacept in early and established RA.

## Introduction

In rheumatoid arthritis (RA), complex co-stimulation-dependent interactions between dendritic cells, T cells, and B cells underlie the generation of an autoimmune response to citrullinated self-proteins. Within the RA synovial membrane and adjacent bone marrow, inflammatory cytokines mediate pro-inflammatory pathways, contributing to the development of chronic disease [1].

Improved understanding of the pathogenesis of RA has led in recent years to the development of new and innovative therapeutic agents with differing mechanisms of action (MoAs), most of which target effector molecules rather than the underlying disease process. The critical role of T cells in RA is widely recognized and, consequently, the modulation of the co-stimulatory signal required for T cell activation is an accepted therapeutic target [2]. To date, abatacept is the only available biologic agent for the treatment of RA that selectively modulates the CD80/CD86:CD28 co-stimulatory signal required for full T cell activation. Abatacept is a fusion protein of the extracellular domain of human cytotoxic T lymphocyte-associated antigen 4 (CTLA-4) linked to the Fc portion of human immunoglobulin (Ig) G1 and competes with CD28 for binding to CD80/CD86. Abatacept is available in intravenous (IV) and subcutaneous (SC) formulations, both of which are licensed globally for the treatment of moderate-to-severe RA. There are much data supporting the efficacy and acceptable safety profile of abatacept in RA, and reviews on both formulations are available [3, 4•, 5, 6].

This review summarizes the latest insights into the MoA of abatacept and provides an overview of recent data regarding the efficacy and safety of abatacept in RA. The potential implications of use early in the disease course are discussed alongside the latest research on biomarkers for predicting response to abatacept treatment.

## Mechanism of action

Full T cell activation requires CD28:CD80/CD86 co-stimulation and triggers cytokine production, clonal expansion, enhanced T cell survival, and the provision of B cell help [7]. In RA, activated CD4+ T cells expressing CD28 significantly infiltrate the synovial membrane of affected joints where they can contribute to the exacerbation of synovitis and joint destruction by secreting inflammatory cytokines and activating synovial cells and osteoclasts. In contrast to, for example, anti-tumor necrosis factor (anti-TNF) agents, abatacept works upstream in the immune response, suppressing the co-stimulatory signal required for naïve T cell activation [7].

### Recent advances in understanding the mechanism of action

Through the selective modulation of the CD28 co-stimulatory pathway or direct binding to CD80 and CD86, abatacept may target additional cell types implicated in the pathogenesis of RA, contributing to the observed clinical effects. The findings of recent in vitro and in vivo studies regarding the biologic effects of abatacept are summarized below.

#### Osteoclasts

**Fig. 1:**
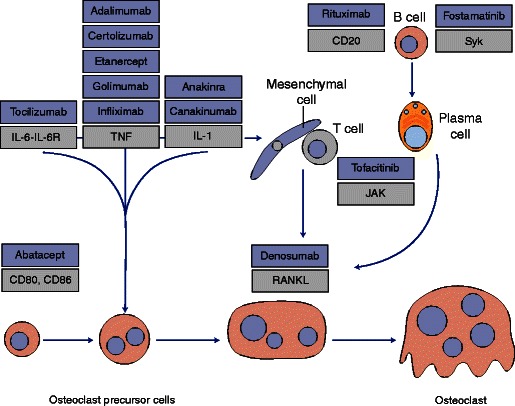
Osteoclast differentiation and bone erosion pathways in rheumatoid arthritis showing the position that different classes of biologic agents exert their major effects [8•]. Anti-rheumatic drugs are shown in *blue boxes*. Abatacept inhibits osteoclast differentiation by directly engaging CD80 and CD86 on the surface of osteoclast precursor cells. Figure reprinted by permission from Macmillan Publishers Ltd: Nat Rev Rheumatol. 2012;8(11):656–64, copyright (2012). *IL* interleukin, *IL-6R* interleukin-6 receptor, *JAK* Janus kinase, *RANKL* receptor activator of nuclear factor κB ligand, *Syk* spleen tyrosine kinase, *TNF* tumor necrosis factor.

Joint erosion and loss of bone mass are characteristic of RA. CD80 and CD86 function as negative regulators for the generation of bone-resorbing osteoclasts, suggesting that abatacept may have direct effects in osteoclast precursors (Fig. 1) [8•]. The stimulation of CD80/CD86 with CTLA-4 activates indoleamine 2,3-dioxygenase in osteoclast precursors, promoting apoptosis [9]. Preclinical studies in C57BL6 mice found that treatment with abatacept was associated with increased bone mineral density, enhanced indices of bone formation, and elevated levels of Wnt10b, the bone anabolic Wnt ligand, in bone marrow [10], suggesting that abatacept may have a positive effect on bone density.

Consistent with the bone loss associated with RA, affected patients may have higher osteoclast precursor frequency than healthy controls [11]. In RA, abatacept treatment has been shown to result in a more pronounced reduction in osteoclast precursor frequency after 4 weeks than methotrexate (MTX) or anti-TNFs, achieving an osteoclast precursor frequency that was indistinguishable from that in healthy controls [9]. In addition, abatacept can significantly reduce A disintegrin and metalloprotease 17 (ADAM17), an enzyme associated with bone and cartilage damage [12]. Together, these findings suggest that abatacept can promote a beneficial balance between bone formation and resorption and may contribute to the termination of inflammatory cytokine networks.

#### Regulatory T cells

Regulatory T cells (Tregs) are necessary to maintain self-tolerance, and failure of Treg activity is believed to play a central role in the development of autoimmune diseases including RA [13•]. Tregs can also protect against TNF-mediated bone loss through the inhibition of osteoclast differentiation [14]. Abatacept has been shown to promote the recovery of Treg cell function in patients with an inadequate response to anti-TNF agents and moderate-to-severe RA [15]. Furthermore, significant decreases in interferon-γ- and interleukin-17-producing T cells and normalization of Treg numbers have been reported in patients with a good European League Against Rheumatism (EULAR) response following abatacept treatment [16]. Thus, abatacept-mediated recovery of Treg inhibitory function may contribute to a reduction in inflammation-induced bone destruction in RA.

#### Effector T cells

A delicate balance exists between T helper (Th)1 and Th17 effector cells and Tregs in the regulation of inflammatory autoimmune disease [17]. Abatacept can modulate T cell effector functions in patients with RA who are anti-citrullinated peptide antibody (ACPA) seropositive. Indeed, treatment with abatacept has been found to lead to a general decrease in effector T cell subsets and a reduction in Th1, Th2, and Th17 cytokines in peripheral blood and cell culture supernatants [18]. CD28− T cells with characteristics of cytotoxic memory T cells are believed to be pathologically relevant in a subgroup of patients with RA [19]. Following treatment with IV abatacept, a reduction in T cell subsets, including the CD28− subset, was noted in a group of 44 patients, 36 (82 %) of whom were anti-cyclic citrullinated peptide (CCP) seropositive [20], suggesting that abatacept modulates T cell receptor tuning required for the generation of CD28 T cells. Together, these recent findings show that abatacept treatment can directly influence the number, phenotype, and function of effector T cells that contribute to RA.

#### Antigen-presenting cells

Multiple cell types express the co-stimulatory molecules CD80 and CD86 necessary for antigen presentation and may thus be affected by abatacept. The consequences of abatacept binding to CD80/CD86 on antigen-presenting cells (APCs) may not be limited to the inhibition of interaction with CD28 in the context of antigen presentation. Abatacept may also contribute to reduced monocyte migration to the synovial tissue. In peripheral blood samples from patients with RA, treatment with abatacept downregulated adhesion molecule expression on CD14+ monocytes and significantly reduced their migratory capacity [21]. Downregulation of pro-inflammatory cytokine gene expression has been reported following exposure of RA synovial macrophages to abatacept in vitro [22]. A further in vitro study demonstrated that abatacept can block synovial CD4 T cell proliferation induced by thymic stromal lipoprotein-primed myeloid dendritic cells, although the inhibitory capacity of abatacept was reduced in the presence of T cell-activating cytokines [23]. Abatacept treatment also resulted in decreased expression of intracellular adhesion molecule-1 and vascular endothelial growth factor-4 in cultured endothelial cells [24]. Thus, direct effects of abatacept on APCs may contribute to its therapeutic effect in RA.

#### B cells

In RA, B cells contribute to the induction of effector T cell differentiation, the secretion of pro-inflammatory cytokines, and bone homeostasis [25]. Data support a direct effect of abatacept on B cells expressing the CD80 and CD86 co-stimulatory molecules. Treatment with abatacept plus MTX was associated with decreased cell proliferation in the RA synovium versus MTX alone and with decreased synovial expression of B cell markers [26]. Abatacept may also inhibit the phosphorylation of spleen tyrosine kinase (Syk), a key molecule in B cell ACPA production [27]. In a group of 30 patients with RA, those with a clinical response to abatacept therapy experienced significant decreases in levels of IgG, IgA, IgM, and post-switch memory B cells and normalization of free light chains at 6 months and decreases in memory B cell subsets at 12 months [28•]. These findings show that abatacept can restore regulation within the memory B cell compartment and highlight the clinical relevance of B cell–T cell interactions in RA.

## Efficacy and safety in clinical trials

Abatacept was initially licensed as an IV formulation. In clinical trials, IV abatacept provided clinically meaningful and sustained benefits in RA signs and symptoms, structural damage, and physical function in early and established disease. Notably, treatment benefits were reported in a range of patient types, including those who were MTX naïve, and inadequate responders to MTX or anti-TNF agents [5]. In an integrated safety analysis of IV abatacept clinical trials (4,149 patients; 12,132 patient-years of exposure), the short- and long-term abatacept safety profiles were consistent with low incidence rates (per 100 patient-years) of serious infections (2.87), malignancies (0.73; excluding non-melanoma skin cancer [NMSC]), and autoimmune events (2.64) [6].

Subsequently, SC abatacept was shown to have similar short- and long-term efficacy to the IV formulation, with clinical benefits maintained over time [29, 30••]. Safety findings with SC abatacept were consistent with those of the IV formulation. In an integrated safety analysis of SC abatacept clinical trials (1,879 patients, 4,215 patient-years of exposure), there were low incidence rates (per 100 patient-years) of serious infections (1.79), malignancies (0.71; excluding NMSC), autoimmune events (1.99), and injection site reactions (1.72) [3].

### IV abatacept—established efficacy in RA

Data continue to emerge supporting the efficacy and safety of IV abatacept in patients with an inadequate response to MTX. Impact of intravenous *A*batacept on *S*ynovitis, osteitis and *S*tructural damage in patients with rheumatoid arthritis and an inadequate response to m*E*thotrexate: a randomized controlled *T*rial (ASSET) evaluated the impact of abatacept (∼10 mg/kg) plus MTX on pathology evident on magnetic resonance imaging (MRI). Although ASSET did not meet its primary endpoint of mean change from baseline to month 4 in wrist synovitis score, abatacept plus MTX was associated with a trend towards reduced synovitis, osteitis, and minimal deterioration in erosion versus MTX alone over 4 months, demonstrating an early effect of abatacept plus MTX on synovium and bone and confirming its effectiveness in patients with an inadequate response to MTX [31].

Long-term data for abatacept in patients with an inadequate response to MTX were recently reported. Five-year results from the long-term extension of the *A*batacept in *I*nadequate responders to *M*ethotrexate (AIM) trial [32] showed that abatacept (∼10 mg/kg) was well-tolerated, with consistent safety findings over the entire study period. Efficacy was sustained over 5 years, with 45.1 % of patients remaining free from radiographic progression at year 5 [33]. Consistent safety and sustained efficacy for IV abatacept over 7 years were also demonstrated in a long-term extension to a phase IIb study in patients with established RA who were MTX inadequate responders. Of note, some patients demonstrated sustained normalization of physical function and health-related quality of life [34].

The findings from randomized controlled trials of IV abatacept are consistent with emerging data from the real-world setting. *A*bata*C*ep*T I*n r*O*uti*N*e clinical practice (ACTION) was a 2-year, observational, prospective study that included patients who initiated abatacept according to the European Summary of Product Characteristics or the Canadian Product Monograph. Results for the first cohort of enrolled patients (May 2008 to January 2011) show that most patients (*n* = 996; 89.4 %) initiated abatacept after the failure of at least one prior biologic agent. The overall 2-year retention rate was 54.4 % [35]. Corticosteroid dose reduction was possible in some patients who had received IV abatacept [36], and retention rates and effectiveness outcomes were superior when abatacept was initiated earlier in the disease course [37], again supporting early use. In addition, IV abatacept was found to be effective both in combination with conventional synthetic disease-modifying anti-rheumatic drugs (DMARDs) and as monotherapy [38], a finding also seen in a 3-year study of Japanese patients who had an inadequate response to either MTX or biologics [39].

### SC abatacept—equivalence to the IV formulation

In 2014, findings from the long-term extension to the *A*batacept *C*omparison of sub[*QU*]cutaneous versus intravenous in *I*nadequate *R*esponders to methotrexat*E* (ACQUIRE) trial, which originally demonstrated comparable efficacy and safety of SC abatacept to the IV formulation, were reported. In the initial study, high retention rates (94.2 %) and low immunogenicity were observed over 6 months for SC abatacept [29]. In the long-term extension, patients who completed the double-blind period received SC abatacept (125 mg/week). Long-term treatment was well tolerated, with no increase in the incidence of safety events over time. Clinical benefits achieved with abatacept in the 6-month double-blind period were maintained over 981 days, including in patients who had switched from the IV to SC formulation [30••].

Common among biologic therapies, immunogenicity and its potential impact on drug efficacy and safety remain a concern for abatacept. The safety, efficacy, and immunogenicity of SC abatacept were assessed in the *A*bata*C*ept in subje*C*ts with rheumat*O*id arthritis ad*M*inistered *P*lus or minus background MTX subcut*AN*eousl*Y* (ACCOMPANY) trial, in which patients received SC abatacept, with or without MTX, without an IV loading dose. During the 4-month short-term period, 3.9 and 4.1 % of patients who received abatacept plus MTX or abatacept monotherapy, respectively, developed transient immunogenicity. During the long-term extension, in which patients receiving abatacept monotherapy could add MTX, one patient developed immunogenicity. The efficacy and safety profile of SC abatacept was consistent with previous clinical experience, and immunogenicity was not associated with loss of efficacy or safety [40]. SC abatacept also had an acceptable safety profile, with low rates of immunogenicity in a long-term extension study in Japanese patients and no changes in safety profile, efficacy, or pharmacokinetics in patients who had developed anti-abatacept antibodies [41].

### Recent advances—abatacept in early RA

#### The possibility of altering the RA disease course

The unique, upstream MoA of abatacept suggests the possibility of altering the disease course through inhibition of the underlying immune response in the early stages of RA. As with many chronic, progressive diseases, a cure for RA remains elusive, and patients face long-term therapy of variable efficacy and associated side effects. In the absence of curative treatment, the option of dose reduction or treatment holidays may be highly desirable.

Several years ago, results from the *A*batacept study to *D*etermine the effectiveness in preventing the development of rheumatoid arthritis in patients with *U*ndifferentiated inflammatory arthritis and to evaluate *S*afety and *T*olerability (ADJUST) highlighted the possibility of altering RA progression by modulating T cell responses through the introduction of abatacept therapy early in the disease course [42]. Expanding on this concept, abatacept dose reduction was assessed in a substudy of the *A*batacept study to *G*auge *R*emission and joint damage progression in methotrexate naïve patients with *E*arly *E*rosive rheumatoid arthritis (AGREE) (mean disease duration = 2.3 years) and poor prognosis. Patients who achieved Disease Activity Score (DAS)28 (erythrocyte sedimentation rate [ESR]) <2.6 following 2 years of IV abatacept (∼10 mg/kg) plus MTX were randomized to continue their current dose of abatacept (*n* = 58) or to receive a reduced dose of ∼5 mg/kg (*n* = 50). The percentage of patients who experienced a protocol-defined relapse over 12 months was similar between the ∼10- and ∼5-mg/kg groups (31 vs. 34 %; hazard ratio = 0.87; 95 % confidence interval [CI], 0.45 to 1.69) [43], suggesting that abatacept dose reduction is possible without increasing the risk of relapse in some patients. Importantly, safety findings were comparable between the two dose groups. Sustained biologic-free remission after 52 weeks was also reported in patients with established RA who attained DAS28 (C-reactive protein [CRP]) <2.3 after two or more years of treatment with abatacept and was most likely in patients with lower Health Assessment Questionnaire-Disability Index (HAQ-DI) or CRP at enrollment [44].

#### Treatment withdrawal in early RA: the AVERT trial

Building on the potential for dose reduction and biologic-free remission in RA, the AVERT trial set out to establish if complete drug-free remission was possible in patients who achieved clinical remission after 1 year of abatacept treatment. Importantly, patients had early RA, and therefore, the impact of early treatment with a selective T cell co-stimulation modulation agent on disease progression could be assessed.

**Fig. 2:**
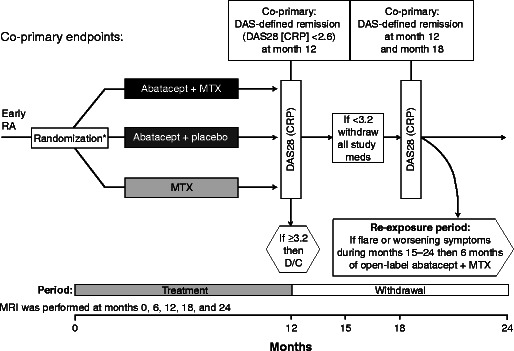
AVERT trial design [45••]. *Asterisk*: Randomization stratified by corticosteroid use at baseline. Figure from Emery P, et al. Ann Rheum Dis. 2015;74(1):19–26, Open Access article distributed in accordance with the Creative Commons Attribution Non Commercial (CC BY-NC 4.0) license, published by the BMJ Group. *AVERT A*ssessing *V*ery *E*arly *R*heumatoid arthritis *T*reatment, *CRP* C-reactive protein, *DAS* Disease Activity Score, *D/C* discontinue, *MRI* magnetic resonance imaging, *MTX* methotrexate, *RA* rheumatoid arthritis.

The AVERT trial was a phase IIIb, multicenter, randomized, active controlled trial [45••] in which patients with <2 years of RA symptoms, DAS28 (CRP) ≥3.2, and anti-CCP2 positivity who were MTX naïve were randomized (1:1:1) to SC abatacept 125 mg/week plus MTX, abatacept monotherapy, or MTX alone for 12 months. Patients who achieved DAS28 (CRP) ≤3.2 at month 12 were eligible to enter a 12-month withdrawal period: abatacept was stopped immediately and MTX and steroids were tapered over 1 month. After month 15, patients who experienced a protocol-defined flare were eligible to enter a re-exposure period with open-label SC abatacept 125 mg/week plus MTX (Fig. 2). Co-primary endpoints were the percentage of randomized and treated patients with DAS28 (CRP) <2.6 at month 12 and at both months 12 and 18, for abatacept plus MTX versus MTX alone.

After 12 months of treatment, a significantly higher proportion of patients in the abatacept plus MTX arm than in the MTX alone arm achieved DAS28 (CRP) <2.6 (70/115 [60.9 %] vs. 52/115 [45.2 %]; odds ratio [OR], 2.01; 95 % CI, 1.18 to 3.43; *p* = 0.010). Furthermore, the proportion who remained in remission at both months 12 and 18 (i.e., after withdrawal of all therapy) was also higher with abatacept plus MTX than with MTX alone (17/115 [14.8 %] vs. 9/115 [7.8 %]; OR, 2.51; 95 % CI, 1.02 to 6.18; *p* = 0.045). The achievement of sustained remission following treatment withdrawal was also seen when using a more stringent cutoff of DAS28 (CRP) <2.4 [46]. Thus, drug-free remission was achieved in a proportion of patients, demonstrating proof of concept that the unique upstream mechanism of abatacept influences the disease process and may allow the removal of drug therapy upon achievement of remission. The benefits extended to notable improvements in fatigue, physical function, pain, and participation in daily activities on treatment [47] and the reduction of MRI-detected joint damage and inflammation, which was maintained for at least 6 months following treatment withdrawal in patients who were in remission or had low disease activity [48]. The safety profiles of both abatacept treatment arms were comparable with MTX, with a similar occurrence of adverse events (AEs) and serious adverse events (SAEs): abatacept plus MTX, 84.9 and 6.7 %, respectively; abatacept monotherapy, 80.2 and 12.1 %, respectively; and MTX, 82.8 and 7.8 %, respectively.

#### Does the AVERT trial have the potential to reshape thinking about the management of RA?

The AVERT trial demonstrated the efficacy of abatacept in patients with early RA, high disease activity, and poor prognostic factors in a population naïve to MTX and highlighted the possibility of substantially altering the disease course with early treatment. In this innovative study, the attainment of sustained remission following withdrawal of all RA therapy in a number of patients, in a population with highly active disease, was suggestive of an underlying effect of co-stimulation modulation on pathogenic autoimmune processes. The removal of all RA therapy may not be a situation anticipated in clinical practice but indicates that initiating early therapy with a drug that acts upstream in the disease process could change the course of RA. These findings will be of particular importance to clinicians if it is possible to identify those patients in whom sustained and complete drug-free remission can be achieved. To this end, efforts have been made to identify predictors of response. In the AVERT trial, less severe disease activity at baseline and longer duration with DAS28 (CRP) <2.6 on treatment were predictors for DAS28 (CRP) <2.6 following treatment withdrawal [49]. Remission is an established treatment target in RA [50••]; therefore, early treatment with biologic agents may be an important step towards achieving sustained remission, particularly in light of the window of opportunity to alter the disease pathway in early RA [51].

#### Co-stimulation modulation in preclinical RA

The attainment of drug-free remission in some patients with early RA raises the possibility of altering the disease course prior to overt clinical disease. Early disease pathway modification will be assessed in a population with preclinical RA, considered at risk of progressive disease, in the *A*rthritis *P*revention in the *P*re-Cl*i*nical *P*hase of *R*A with *A*batacept (APPIPRA) trial (EUDraCT, 2013-003413-18). Enrolled patients will be seropositive for ACPA and rheumatoid factor (RF), DMARD naïve, and have joint pain but no clinical synovitis. Patients will be randomized to SC abatacept 125 mg/week or placebo for 52 weeks. The primary outcome measure is the time to development of three or more swollen joints or 2010 American College of Rheumatology (ACR)/EULAR criteria for RA [52].

### Recent advances—abatacept versus anti-TNF agents

**Fig. 3:**
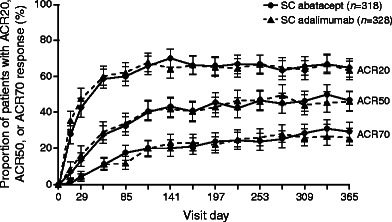
Percentages of patients with rheumatoid arthritis who achieved ACR20, ACR50, and ACR70 responses over 1 year by treatment group in the AMPLE trial [53]. Intent-to-treat population. *Error bars* show 95 % confidence intervals. Reprinted from Weinblatt ME, et al. Arthritis Rheum. 2013;65(1):28–38. © 2013, American College of Rheumatology. ACR American College of Rheumatology, *AMPLE A*batacept versus adali*M*umab com*P*arison in bio*L*ogic-naïv*E* rheumatoid arthritis subjects with background methotrexate, *SC* subcutaneous.

Abatacept is approved in moderate-to-severe RA after failure of MTX or an anti-TNF agent. Anti-TNF agents are well established as first-line biologic therapy in patients with RA. Consequently, clinicians are faced with an increasing choice of possible biologic agents in patients with an inadequate response to MTX and little data to guide treatment decisions. The *A*batacept versus adali*M*umab com*P*arison in bio*L*ogic-naïv*E* rheumatoid arthritis subjects with background methotrexate (AMPLE) trial was a head-to-head comparison of SC abatacept versus adalimumab in patients with active RA who were biologic naïve and had an inadequate response to MTX and was the first direct comparison of two biologic DMARDs with differing MoAs in patients receiving background MTX. Patients were randomized (1:1) to SC abatacept 125 mg/week (*n* = 318) or SC adalimumab 40 mg biweekly (*n* = 328), both with MTX, for 2 years of blinded therapy. Abatacept was shown to be non-inferior to adalimumab across multiple efficacy measures, including the primary endpoint of ACR20 response at 1 year (64.8 vs. 63.4 %). Kinetics of response were similar between the two treatments, as reflected in ACR20/50/70 response rates (Fig. 3), mean DAS28 (CRP), and percentages of patients with HAQ-DI response over time [53]. Over 2 years, abatacept and adalimumab were similarly effective based on clinical and functional outcomes, including ACR20/50/70 response rates, HAQ-DI response, the proportion of patients achieving DAS28 (CRP) <2.6, Clinical Disease Activity Index or Simplified Disease Activity Index remission [54], and patient-reported outcomes [55]. Clinical response was comparable between treatments regardless of disease duration [56]. Abatacept and adalimumab were similarly effective at inhibiting radiographic progression: 84.8 and 83.8 % of patients, respectively, were classified as radiographic non-progressors at 2 years (change from baseline in total Sharp score ≤2.2 [smallest detectable change]) [57••]. Remission, low disease activity, DAS28 (CRP) <2.6, and DAS28 (CRP) ≤3.2 were highly correlated with the prevention of radiographic progression at 2 years for both abatacept and adalimumab [58]. Equivalent efficacy of abatacept and adalimumab was seen despite differences in MoAs, as indicated by gene expression analysis. In the AMPLE trial, abatacept was more selective than adalimumab in modulating gene expression after 3 months of treatment [59]. The observed differences in treatment- and response-dependent clusters further highlight the different MoAs of abatacept and adalimumab leading to similar clinical outcomes.

The results of the AMPLE trial have implications for clinical practice and suggest that abatacept and adalimumab should be considered equally effective, with a similar onset of action in the treatment of RA in patients with an inadequate response to MTX. Importantly, cumulative rates of AEs and SAEs over 2 years were similar for abatacept and adalimumab (92.8 vs. 91.5 % and 13.8 vs. 16.5 %, respectively), but discontinuations due to AEs and SAEs were more common with adalimumab (3.8 vs. 5.8 % and 1.6 vs. 4.9 %, respectively). Additionally, there were fewer serious infections and injection site reactions with abatacept versus adalimumab (3.8 vs. 5.8 % [including two cases of tuberculosis with adalimumab] and 4.1 vs. 10.4 %, respectively) [57••].

The results of the AMPLE trial confirm, in a head-to-head study, earlier findings from *A*batacept or infliximab versus placebo, a *T*rial for *T*olerability, *E*fficacy and *S*afety in *T*reating rheumatoid arthritis (ATTEST), which had suggested equivalent efficacy for abatacept and an anti-TNF therapy (infliximab). In ATTEST, patients were randomized (3:3:2) to IV abatacept (∼10 mg/kg every 4 weeks), infliximab (3 mg/kg every 8 weeks), or placebo, all with MTX. Abatacept and infliximab demonstrated similar efficacy over 12 months and abatacept had a relatively more acceptable safety and tolerability profile compared with infliximab [60].

A pooled analysis using data from the AMPLE trial and ATTEST strongly suggested that abatacept plus MTX was comparable to adalimumab and infliximab for the reduction of disease activity in patients with an inadequate response to MTX and active RA [61]. This analysis was extended to cover autoimmunity and showed that the anti-TNF therapies were associated with greater autoantibody induction than abatacept in both studies (Table 1), implying that abatacept can result in blockade of B cell function and autoantibody production [62].

**Table 1:** Percentage of patients with ANA and anti-dsDNA autoantibody seroconversion in the ATTEST and AMPLE trials [62]

			Seropositive (baseline negative to post-baseline positive)		Seronegative (baseline positive to post-baseline negative)	
ATTEST			IV ABA + MTX	IFX + MTX	IV ABA + MTX	IFX + MTX
ANA	DB period^a^	Month 6	1.7 (2/115)	32.2 (38/118)	37.5 (12/32)	22.2 (8/36)
		Year 1	6.5 (7/107)	47.7 (51/107)	46.7 (14/30)	11.4 (4/35)
	OLE^b^		ABA-to-ABA	IFX-to-ABA	ABA-to-ABA	IFX-to-ABA
		Year 1 (baseline)	6.1 (6/98)	48.5 (48/99)^c^	48.0 (12/25)	12.1 (4/33)^c^
		Year 2	14.6 (14/96)	22.4 (22/98)^c^	40.7 (11/27)	20.6 (7/34)^c^
Anti-dsDNA	DB period^a^	Month 6	0.8 (1/128)	38.6 (51/132)	20.0 (2/10)	21.4 (3/14)
		Year 1	2.4 (3/127)	47.7 (61/128)	25.0 (2/8)	7.1 (1/14)
	OLE^b^		ABA-to-ABA	IFX-to-ABA	ABA-to-ABA	IFX-to-ABA
		Year 1 (baseline)	2.5 (3/118)	48.3 (57/118)^c^	16.7 (1/6)	7.1 (1/14)^c^
		Year 2	2.6 (3/114)	13.3 (15/113)^c^	37.5 (3/8)	33.3 (5/15)^c^
AMPLE			SC ABA + MTX	ADA + MTX	SC ABA + MTX	ADA + MTX
ANA		Year 1	5.2 (12/229)	13.3 (28/210)	31.9 (23/72)	18.1 (17/94)
		Year 2	6.3 (12/190)	14.7 (24/163)	45.0 (27/60)	18.5 (15/81)
Anti-dsDNA		Year 1	0.3 (1/299)	9.9 (29/293)	100.0 (1/1)	60.0 (3/5)
		Year 2	0 (0/248)	12.2 (29/237)	100.0 (1/1)	75.0 (3/4)

Reproduced from Buch H, et al. Ann Rheum Dis. 2015;74(Suppl 2):1053–4, copyright 2015, with permission from BMJ Publishing Group Ltd. Data are % (*n*/*N*)

*ABA* abatacept, *ADA* adalimumab, *AMPLE A*batacept versus adali*M*umab com*P*arison in bio*L*ogic-naïv*E* rheumatoid arthritis subjects with background methotrexate, *ANA* antinuclear antibodies, *anti-dsDNA* anti-double-stranded deoxyribonucleic acid, *ATTEST A*batacept or infliximab versus placebo, a *T*rial for *T*olerability, *E*fficacy and *S*afety in *T*reating rheumatoid arthritis, *DB* double blind, *IFX* infliximab, *IV* intravenous, *MTX* methotrexate, *OLE* open-label long-term extension, *SC* subcutaneous

^a^Intent-to-treat population

^b^Only patients who entered the OLE

^c^Patients switched to IV ABA + MTX

These findings showing equivalent efficacy for abatacept and anti-TNF agents in patients with an inadequate response to MTX, together with results from the AVERT trial, support the early use of abatacept, prior to the initiation of anti-TNF therapy. Real-world data are also emerging that support the use of abatacept as a first-line biologic agent in RA and demonstrate that early treatment can lead to improvements in disease activity [63] and physical function [64]. Furthermore, Japanese registry data show that there may also be comparable efficacy of abatacept and anti-TNF agents in patients with established RA and high disease activity [65].

## Predicting response to abatacept—recent biomarker studies

Identifying patients who are most likely to respond to a particular treatment would greatly aid clinicians in making treatment decisions. It is becoming increasingly apparent that ACPA status may predict treatment response to abatacept. Data from the AVERT trial show that abatacept impacts the maturation of ACPA response in early RA: the concentration of ACPA IgG, IgM, and IgA isotypes and the average number of epitopes recognized were reduced by a substantially greater extent with abatacept plus MTX versus abatacept monotherapy or MTX alone over 1 year [66]. Such improvements in ACPA profile may contribute positively to clinical outcomes and identify responders.

Despite obvious clinical utility, reliable biomarkers for predicting treatment response remain elusive. In the AVERT trial, the clinical efficacy of abatacept plus MTX was greater in patients who were anti-CCP2 IgM positive versus negative and in patients who did versus did not seroconvert over time [67]. In the AMPLE trial, higher anti-CCP2 antibody titer at baseline correlated with greater efficacy in patients treated with abatacept but not with adalimumab [68]. A similar trend was seen for patient-reported outcomes, including patient global assessment, pain, and Short-Form-36 Health Survey physical component summary score [69]. These findings are supported by real-world studies wherein baseline anti-CCP seropositivity or double anti-CCP and RF seropositivity were identified as predictors of response and/or higher treatment retention of abatacept [70–72]. Notably, these findings contrast with a large meta-analysis (14 studies; 5,561 patients) that failed to identify an association between clinical response to anti-TNF treatment and RF or anti-CCP antibody status [73], highlighting the possibility of novel predictors of treatment response for abatacept.

## Pipeline co-stimulation agents

To date, abatacept is the first and only selective T cell co-stimulation modulation agent approved for the treatment of moderate-to-severe RA. Although agents that target co-stimulation are in development, I am unaware of any other agents with this MoA that are currently undergoing clinical trials in RA.

## Conclusions

Abatacept is the only approved agent for use in moderate-to-severe RA that modulates T cell co-stimulation. Upstream modulation in the RA disease pathway contributes to multiple downstream effects, and recent evidence suggests that abatacept may work across multiple pathways of the RA disease process, impacting many cell types. In recent years, clinical trials have demonstrated that IV abatacept is an effective and well-tolerated treatment in patients with an inadequate response to MTX or anti-TNF agents, and the SC formulation is similarly effective in patients with an inadequate response to MTX. Data from interventional and observational clinical trials that support the long-term safety and efficacy of abatacept continue to accumulate. The AVERT trial showed that early treatment with abatacept has the potential to alter the RA disease course in some patients, allowing for complete withdrawal of all therapy without relapse. In the AMPLE trial, abatacept was found to have equivalent efficacy, onset of action, and inhibitory effect on the progression of structural damage compared with an anti-TNF agent, suggesting that abatacept and anti-TNF therapies could be considered equally effective in patients with an inadequate response to MTX. Together, these findings place abatacept as an important option for the treatment of early and established RA.
